# 3,6-Didehydro-5-hy­droxy-1,2-*O*-iso­propyl­idene-5-*C*-nitro­meth­yl-α-d-gluco­furan­ose

**DOI:** 10.1107/S160053681102191X

**Published:** 2011-06-18

**Authors:** Qiurong Zhang, Pan Li, Xuebin Chen, Xiandong Wang, Hongmin Liu

**Affiliations:** a New Drug Reseach & Development Center, Zhengzhou Univresity, Zhengzhou 450001, People’s Republic of China

## Abstract

The title compound, C_10_H_15_NO_7_, consists of one methyl­enedi­oxy ring and two fused tetra­hydro­furan rings. The three fused rings exhibit *cis* arrangements at the ring junctions. One O atom of a tetra­hydro­furan ring and the H atoms bound to the neighboring C atoms are disordered over two orientations with site-occupancy factors of 0.69 (1) and 0.31 (1). intra­molecular O—H⋯O and C—H⋯O inter­actions stabilize the mol­ecular conformation. In the crystal structure, inter­molecular O—H⋯O and C—H⋯O inter­actions link the mol­ecules into a three-dimensional network.

## Related literature

For the synthesis of aza­sugars, see: Choi *et al.* (1991 ▶); Kvaernø *et al.* (2001 ▶). For the Henry reaction used to obtain the title compound, see: Saito *et al.* (2002 ▶). For research on carbohydrates and aza­sugars, see: Liu *et al.* (2004 ▶); Ke *et al.* (2009 ▶); Zhang *et al.* (2011 ▶). For a similar structure, see: Zhang & Yang (2010 ▶).
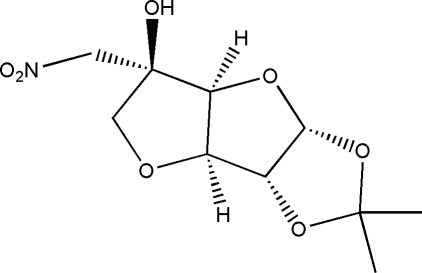


## Experimental

### 

#### Crystal data

C_10_H_15_NO_7_
*M* *_r_* = 261.23
Orthorhombic, 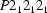

*a* = 5.63290 (13) Å
*b* = 8.36405 (15) Å
*c* = 25.4014 (5) Å
*V* = 1196.76 (4) Å^3^
*Z* = 4
Cu *K*α radiation
μ = 1.07 mm^−1^
*T* = 291 K
0.24 × 0.22 × 0.20 mm

#### Data collection

Bruker SMART diffractometer
Absorption correction: multi-scan (*SADABS*; Sheldrick, 1996 ▶) *T*_min_ = 0.783, *T*_max_ = 0.814
7427 measured reflections
2249 independent reflections
2161 reflections with *I* > 2σ(*I*)
*R*_int_ = 0.021

#### Refinement

*R*[*F* ^2^ > 2σ(*F* ^2^)] = 0.053
*wR*(*F* ^2^) = 0.165
*S* = 1.08
2249 reflections
174 parameters
8 restraints
H atoms treated by a mixture of independent and constrained refinement
Δρ_max_ = 0.59 e Å^−3^
Δρ_min_ = −0.43 e Å^−3^

### 

Data collection: *SMART* (Bruker, 2007 ▶); cell refinement: *SAINT* (Bruker, 2007 ▶); data reduction: *SAINT*; program(s) used to solve structure: *SHELXS97* (Sheldrick, 2008 ▶); program(s) used to refine structure: *SHELXL97* (Sheldrick, 2008 ▶); molecular graphics: *SHELXTL* (Sheldrick, 2008 ▶); software used to prepare material for publication: *SHELXL97*.

## Supplementary Material

Crystal structure: contains datablock(s) I, global. DOI: 10.1107/S160053681102191X/zq2104sup1.cif

Structure factors: contains datablock(s) I. DOI: 10.1107/S160053681102191X/zq2104Isup2.hkl

Additional supplementary materials:  crystallographic information; 3D view; checkCIF report

## Figures and Tables

**Table 1:** Hydrogen-bond geometry (Å, °)

*D*—H⋯*A*	*D*—H	H⋯*A*	*D*⋯*A*	*D*—H⋯*A*
O5—H5⋯O4	0.86 (6)	2.13 (6)	2.696 (3)	123 (5)
O5—H5⋯O3^i^	0.86 (6)	2.24 (6)	2.703 (3)	114 (4)
C1—H1⋯O5^ii^	0.98	2.48	3.371 (3)	152
C4—H4⋯O6	0.98	2.39	3.074 (4)	126

Symmetry codes: (i) 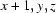; (ii) 
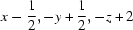
.

**Fig. 1.**
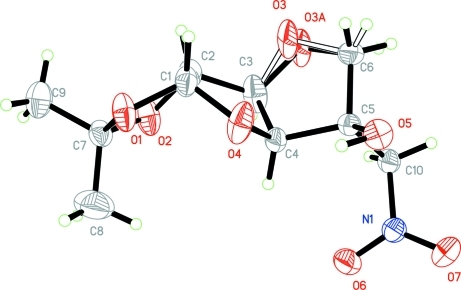
The molecular structure of the title compound showing the atomic numbering and 30% probability displacement ellipsoids.

## supplementary crystallographic information

### Comment

Azasugars containing some novel glycosyls such as bicyclo-glycosyl and heterocycle glycosyl, the synthesis of which being known for many years (Choi *et al.*, 1991; Kvaernø *et al.*, 2001), have attracted a growing interest due to their potent antiviral activity. As a contribution to the research for carbohydrate and azasugars compounds (Liu *et al.*, 2004; Ke *et al.*, 2009), we report here the synthesis and X-ray crystal structure of the title compound, an intermediate of bicyclo-glycosyl. The title compound, which shows a similar structure to the one previously reported by Zhang & Yang (2010), was enantiomerically synthesized at room tempeature by means of the Henry reaction (Saito *et al.*, 2002).

The title compound, C_10_H_15_NO_7_, consists of one methylenedioxy ring and two fused tetrahydrofuran rings. The three fused rings exhibit *cis* arrangements at the ring junctions and give two V-shaped molecules. One O atom of a tetrahydrofuran ring moiety is disordered over two positions with site-occupancy factors of 0.69 (1) and 0.31 (1), the H atoms bound to the neighboring C atoms were disordered as well. The bond angles O2—C7—O1 and C8—C7—C9 around the isopropylidene are 105.6 (2) and 113.2 (3)°, which are almost equal to the corresponding bond angles reported by Zhang & Yang (2010). The bond angle O5—C5—C10 containing simultaneously hydroxy and nitromethylene is 108.3 (2)°. The torsion angles C2—C3—C4—C5, O3—C3—C4—O4, O4—C1—C2—O2 and O1—C1—C2—C3 are -140.4 (3), 99.7 (4), 98.6 (3) and -135.2 (3) °, respectively.

In the crystal structure, some intra- and intermolecular O—H···O and C—H···O interactions exist to stabilize the molecular conformation and link the molecules into a three-dimensional network.

### Experimental

The title compound was synthesized from 3,6-didehydro-1,2-*O*-isopropylidene-5-carbonyl-α-*D*-glucofuranose with Henry reaction as described previously by Saito *et al.* (2002) whose starting material was D-glucose. To a solution of the starting material (2.4 g, 9.2 mmol) in tetrahydrofuran (30 ml) was added CH_3_NO_2_ (0.82 ml) and potassium fluoride (0.84 g) under ice bath. The mixture was stirred at room temperature for 12 h. After the material was consumed, the reaction mixture was filtered to remove the KF. The filtrate was concentrated *in vacuo* to yield the residue, which was recrystalized in CH_3_OH to obtain the title compound as a white solid. Crystals suitable for X-ray analysis were grown by slow evaporation from methanol at room temperature for two weeks.

### Refinement

All H atoms were placed geometrically and treated as riding on their parent atoms, with C—H = 0.96 Å and *U*_iso_(H) =1.5*U*_eq_(C) for methyl H atoms, with C—H = 0.97 Å and *U*_iso_(H) =1.2*U*_eq_(C) for methylene H atoms, and with C—H = 0.98 Å and *U*_iso_(H) =1.2*U*_eq_(C) for methine H atoms. The hydroxy H atom was freely refined. In the absence of any significant anomalous scatterers in the molecule, attempts to confirm the absolute structure by refinement of the Flack parameter in the presence of 896 sets of Friedel equivalents led to an inconclusive value of 0.0 (3). Therefore, the Friedel pairs were merged before the final refinement and the absolute configuration was assigned to correspond with that of the known chiral centres in the precursor molecule, which remained unchanged during the synthesis of the title compound.

One O atom of a tetrahydrofuran ring moiety is disordered over two positions with site-occupancy factors of 0.69 (1) and 0.31 (1), the H atoms bound to the neighboring atoms C3 and C6 were disordered as well over two positions.

### Crystal data

**Table:**

C_10_H_15_NO_7_	*D*_x_ = 1.450 Mg m^−^^3^
*M**_r_* = 261.23	Melting point = 392–394 K
Orthorhombic, *P*2_1_2_1_2_1_	Cu *K*α radiation, λ = 1.54184 Å
Hall symbol: P 2ac 2ab	Cell parameters from 5453 reflections
*a* = 5.63290 (13) Å	θ = 3.5–70.0°
*b* = 8.36405 (15) Å	µ = 1.07 mm^−^^1^
*c* = 25.4014 (5) Å	*T* = 291 K
*V* = 1196.76 (4) Å^3^	Block, white
*Z* = 4	0.24 × 0.22 × 0.20 mm
*F*(000) = 552	

### Data collection

**Table:**

Bruker SMART diffractometer	2249 independent reflections
Radiation source: Enhance (Cu) X-ray Source	2161 reflections with *I* > 2σ(*I*)
graphite	*R*_int_ = 0.021
Detector resolution: 0 pixels mm^-1^	θ_max_ = 70.2°, θ_min_ = 3.5°
ω scans	*h* = −6→4
Absorption correction: multi-scan (*SADABS*; Sheldrick, 1996)	*k* = −9→10
*T*_min_ = 0.783, *T*_max_ = 0.814	*l* = −29→30
7427 measured reflections	

### Refinement

**Table:**

Refinement on *F*^2^	Primary atom site location: structure-invariant direct methods
Least-squares matrix: full	Secondary atom site location: difference Fourier map
*R*[*F*^2^ > 2σ(*F*^2^)] = 0.053	Hydrogen site location: inferred from neighbouring sites
*wR*(*F*^2^) = 0.165	H atoms treated by a mixture of independent and constrained refinement
*S* = 1.08	*w* = 1/[σ^2^(*F*_o_^2^) + (0.1069*P*)^2^ + 0.3286*P*] where *P* = (*F*_o_^2^ + 2*F*_c_^2^)/3
2249 reflections	(Δ/σ)_max_ < 0.001
174 parameters	Δρ_max_ = 0.59 e Å^−^^3^
8 restraints	Δρ_min_ = −0.43 e Å^−^^3^

### Special details

**Table:**

Experimental. Melting point: 119–121 °C; *R*_f_ = 0.67 (CHCl_3_/EtOAc, 7:3); ^1^H NMR (400 MHz, CDCl_3_) σ: 5.98 (d, *J* = 3.4 Hz, 1H), 4.81 (d, *J* = 4.3 Hz, 1H), 4.68 (d, *J* = 3.4 Hz, 1H), 4.60 (dd, *J* = 8.3, 4.0 Hz, 2H), 4.52 (d, *J* = 12.3 Hz, 1H), 3.83 (d, *J* = 10.0 Hz, 1H), 3.75 (d, *J* = 10.0 Hz, 1H), 3.36 (s, 1H), 1.51 (s, 3H), 1.36 (s, 3H); ^13^C NMR (100 MHz, CDCl_3_) σ: 113.60, 107.18, 85.35, 85.08, 83.80, 78.62, 78.42, 74.89, 27.49, 26.77.
Geometry. All e.s.d.'s (except the e.s.d. in the dihedral angle between two l.s. planes) are estimated using the full covariance matrix. The cell e.s.d.'s are taken into account individually in the estimation of e.s.d.'s in distances, angles and torsion angles; correlations between e.s.d.'s in cell parameters are only used when they are defined by crystal symmetry. An approximate (isotropic) treatment of cell e.s.d.'s is used for estimating e.s.d.'s involving l.s. planes.
Refinement. Refinement of *F*^2^ against ALL reflections. The weighted *R*-factor *wR* and goodness of fit *S* are based on *F*^2^, conventional *R*-factors *R* are based on *F*, with *F* set to zero for negative *F*^2^. The threshold expression of *F*^2^ > σ(*F*^2^) is used only for calculating *R*-factors(gt) *etc*. and is not relevant to the choice of reflections for refinement. *R*-factors based on *F*^2^ are statistically about twice as large as those based on *F*, and *R*- factors based on ALL data will be even larger.

### Fractional atomic coordinates and isotropic or equivalent isotropic displacement parameters (Å^2^)

**Table:**

	*x*	*y*	*z*	*U*_iso_*/*U*_eq_	Occ. (<1)
O1	0.8215 (5)	−0.0074 (2)	0.86303 (8)	0.0635 (6)	
O2	0.5738 (5)	0.1567 (3)	0.81809 (9)	0.0693 (7)	
O3	0.4254 (5)	0.4145 (8)	0.92867 (15)	0.0833 (16)	0.695 (10)
O3A	0.4631 (15)	0.3468 (10)	0.93694 (15)	0.0833 (16)	0.305 (10)
O4	0.8877 (4)	0.2199 (2)	0.91272 (11)	0.0726 (8)	
O5	1.0074 (4)	0.4587 (3)	0.97889 (8)	0.0526 (5)	
H5	1.070 (10)	0.374 (6)	0.966 (2)	0.113 (19)*	
O6	1.1140 (5)	0.6590 (3)	0.86098 (10)	0.0729 (7)	
O7	1.2025 (5)	0.7827 (3)	0.93199 (11)	0.0735 (7)	
N1	1.0673 (5)	0.7059 (3)	0.90501 (9)	0.0487 (6)	
C1	0.7251 (6)	0.1003 (3)	0.89903 (10)	0.0530 (7)	
H1	0.6693	0.0440	0.9305	0.064*	
C2	0.5231 (5)	0.1803 (4)	0.87177 (13)	0.0598 (8)	
H2	0.3681	0.1375	0.8822	0.072*	
C3	0.5524 (6)	0.3547 (5)	0.88496 (14)	0.0712 (11)	
H3A	0.5120	0.4186	0.8539	0.085*	0.695 (10)
H3B	0.4794	0.4325	0.8611	0.085*	0.305 (10)
C4	0.8110 (5)	0.3724 (3)	0.89597 (11)	0.0450 (6)	
H4	0.9001	0.4120	0.8655	0.054*	
C5	0.8201 (4)	0.4884 (3)	0.94342 (9)	0.0378 (5)	
C6	0.5819 (5)	0.4568 (4)	0.96985 (11)	0.0517 (6)	
H6A	0.5953	0.3704	0.9952	0.062*	0.695 (10)
H6B	0.5259	0.5518	0.9879	0.062*	0.695 (10)
H6C	0.6048	0.4120	1.0047	0.062*	0.305 (10)
H6D	0.4915	0.5550	0.9730	0.062*	0.305 (10)
C7	0.7269 (6)	0.0235 (3)	0.81191 (10)	0.0488 (7)	
C8	0.9245 (10)	0.0693 (8)	0.7760 (2)	0.1050 (16)	
H8A	1.0428	−0.0133	0.7761	0.157*	
H8B	0.9940	0.1677	0.7879	0.157*	
H8C	0.8640	0.0830	0.7410	0.157*	
C9	0.5948 (9)	−0.1217 (5)	0.79390 (18)	0.0830 (12)	
H9A	0.4567	−0.1366	0.8155	0.125*	
H9B	0.6958	−0.2138	0.7967	0.125*	
H9C	0.5471	−0.1080	0.7579	0.125*	
C10	0.8305 (5)	0.6646 (3)	0.92708 (11)	0.0437 (6)	
H10A	0.7090	0.6856	0.9009	0.052*	
H10B	0.7985	0.7315	0.9575	0.052*	

### Atomic displacement parameters (Å^2^)

**Table:**

	*U*^11^	*U*^22^	*U*^33^	*U*^12^	*U*^13^	*U*^23^
O1	0.0991 (17)	0.0435 (10)	0.0480 (10)	0.0213 (12)	−0.0142 (12)	−0.0086 (8)
O2	0.0906 (17)	0.0641 (13)	0.0533 (11)	0.0267 (12)	−0.0249 (12)	−0.0142 (10)
O3	0.0301 (13)	0.080 (4)	0.140 (3)	0.0168 (15)	0.0027 (14)	−0.075 (3)
O3A	0.0301 (13)	0.080 (4)	0.140 (3)	0.0168 (15)	0.0027 (14)	−0.075 (3)
O4	0.0591 (13)	0.0422 (10)	0.117 (2)	0.0172 (10)	−0.0355 (13)	−0.0200 (12)
O5	0.0480 (10)	0.0557 (12)	0.0542 (11)	0.0076 (9)	−0.0076 (9)	0.0032 (9)
O6	0.0897 (17)	0.0640 (14)	0.0650 (13)	−0.0128 (13)	0.0300 (13)	−0.0073 (11)
O7	0.0693 (13)	0.0674 (13)	0.0836 (16)	−0.0247 (13)	−0.0016 (13)	−0.0072 (12)
N1	0.0578 (14)	0.0353 (10)	0.0529 (12)	−0.0020 (10)	0.0079 (11)	0.0004 (9)
C1	0.084 (2)	0.0345 (12)	0.0409 (13)	−0.0002 (13)	−0.0035 (14)	−0.0024 (9)
C2	0.0429 (14)	0.0694 (19)	0.0670 (18)	−0.0064 (14)	−0.0001 (13)	−0.0269 (15)
C3	0.0604 (19)	0.073 (2)	0.080 (2)	0.0310 (17)	−0.0269 (17)	−0.0296 (18)
C4	0.0550 (15)	0.0337 (11)	0.0463 (13)	0.0003 (11)	0.0054 (12)	−0.0045 (10)
C5	0.0377 (11)	0.0358 (11)	0.0398 (11)	0.0029 (10)	0.0024 (10)	0.0019 (9)
C6	0.0494 (14)	0.0501 (15)	0.0556 (14)	−0.0030 (12)	0.0124 (12)	−0.0011 (12)
C7	0.0614 (16)	0.0471 (14)	0.0380 (12)	0.0021 (12)	−0.0008 (12)	−0.0023 (10)
C8	0.100 (3)	0.133 (4)	0.082 (3)	−0.014 (3)	0.033 (3)	0.005 (3)
C9	0.096 (3)	0.074 (2)	0.079 (2)	−0.011 (2)	−0.014 (2)	−0.0243 (19)
C10	0.0494 (13)	0.0330 (11)	0.0487 (12)	0.0056 (10)	0.0050 (12)	−0.0011 (10)

### Geometric parameters (Å, °)

**Table:**

O1—C1	1.394 (3)	C3—H3A	0.9800
O1—C7	1.427 (3)	C3—H3B	0.9800
O2—C2	1.407 (4)	C4—C5	1.548 (3)
O2—C7	1.418 (4)	C4—H4	0.9800
O3—C3	1.412 (3)	C5—C6	1.524 (3)
O3—C6	1.413 (3)	C5—C10	1.532 (3)
O3A—C6	1.412 (3)	C6—H6A	0.9700
O3A—C3	1.414 (3)	C6—H6B	0.9700
O4—C1	1.400 (4)	C6—H6C	0.9700
O4—C4	1.413 (3)	C6—H6D	0.9700
O5—C5	1.409 (3)	C7—C8	1.488 (6)
O5—H5	0.86 (6)	C7—C9	1.497 (5)
O6—N1	1.214 (3)	C8—H8A	0.9600
O7—N1	1.209 (3)	C8—H8B	0.9600
N1—C10	1.487 (4)	C8—H8C	0.9600
C1—C2	1.490 (5)	C9—H9A	0.9600
C1—H1	0.9800	C9—H9B	0.9600
C2—C3	1.506 (5)	C9—H9C	0.9600
C2—H2	0.9800	C10—H10A	0.9700
C3—C4	1.491 (4)	C10—H10B	0.9700
			
C1—O1—C7	109.5 (2)	C6—C5—C4	101.8 (2)
C2—O2—C7	109.9 (2)	C10—C5—C4	113.1 (2)
C3—O3—C6	110.8 (2)	O3A—C6—C5	105.6 (3)
C6—O3A—C3	110.7 (3)	O3—C6—C5	105.5 (2)
C1—O4—C4	111.7 (2)	O3A—C6—H6A	86.8
C5—O5—H5	102 (4)	O3—C6—H6A	110.6
O7—N1—O6	123.9 (3)	C5—C6—H6A	110.6
O7—N1—C10	118.3 (2)	O3A—C6—H6B	131.3
O6—N1—C10	117.8 (2)	O3—C6—H6B	110.6
O1—C1—O4	111.7 (3)	C5—C6—H6B	110.6
O1—C1—C2	106.4 (2)	H6A—C6—H6B	108.8
O4—C1—C2	107.1 (2)	O3A—C6—H6C	110.6
O1—C1—H1	110.5	O3—C6—H6C	131.4
O4—C1—H1	110.5	C5—C6—H6C	110.6
C2—C1—H1	110.5	H6B—C6—H6C	85.9
O2—C2—C1	103.5 (2)	O3A—C6—H6D	110.6
O2—C2—C3	109.2 (3)	O3—C6—H6D	86.9
C1—C2—C3	104.4 (2)	C5—C6—H6D	110.6
O2—C2—H2	113.0	H6A—C6—H6D	128.1
C1—C2—H2	113.0	H6C—C6—H6D	108.7
C3—C2—H2	113.0	O2—C7—O1	105.6 (2)
O3—C3—C4	108.2 (2)	O2—C7—C8	108.7 (3)
O3A—C3—C4	100.2 (4)	O1—C7—C8	108.9 (3)
O3—C3—C2	117.5 (4)	O2—C7—C9	111.7 (3)
O3A—C3—C2	97.1 (4)	O1—C7—C9	108.5 (3)
C4—C3—C2	104.2 (2)	C8—C7—C9	113.2 (3)
O3—C3—H3A	108.9	C7—C8—H8A	109.5
O3A—C3—H3A	134.0	C7—C8—H8B	109.5
C4—C3—H3A	108.9	H8A—C8—H8B	109.5
C2—C3—H3A	108.9	C7—C8—H8C	109.5
O3—C3—H3B	92.3	H8A—C8—H8C	109.5
O3A—C3—H3B	117.4	H8B—C8—H8C	109.5
C4—C3—H3B	117.4	C7—C9—H9A	109.5
C2—C3—H3B	117.4	C7—C9—H9B	109.5
O4—C4—C3	105.4 (3)	H9A—C9—H9B	109.5
O4—C4—C5	108.7 (2)	C7—C9—H9C	109.5
C3—C4—C5	103.9 (2)	H9A—C9—H9C	109.5
O4—C4—H4	112.7	H9B—C9—H9C	109.5
C3—C4—H4	112.7	N1—C10—C5	111.1 (2)
C5—C4—H4	112.7	N1—C10—H10A	109.4
O5—C5—C6	110.3 (2)	C5—C10—H10A	109.4
O5—C5—C10	108.3 (2)	N1—C10—H10B	109.4
C6—C5—C10	108.7 (2)	C5—C10—H10B	109.4
O5—C5—C4	114.3 (2)	H10A—C10—H10B	108.0
			
C7—O1—C1—O4	−104.4 (3)	C2—C3—C4—C5	−140.4 (3)
C7—O1—C1—C2	12.2 (3)	O4—C4—C5—O5	34.3 (3)
C4—O4—C1—O1	115.3 (3)	C3—C4—C5—O5	146.2 (3)
C4—O4—C1—C2	−0.9 (4)	O4—C4—C5—C6	−84.7 (3)
C7—O2—C2—C1	22.4 (3)	C3—C4—C5—C6	27.2 (3)
C7—O2—C2—C3	133.2 (3)	O4—C4—C5—C10	158.9 (2)
O1—C1—C2—O2	−20.9 (3)	C3—C4—C5—C10	−89.2 (3)
O4—C1—C2—O2	98.6 (3)	C3—O3A—C6—O3	70.2 (3)
O1—C1—C2—C3	−135.2 (3)	C3—O3A—C6—C5	−23.2 (8)
O4—C1—C2—C3	−15.6 (3)	C3—O3—C6—O3A	−70.5 (3)
C6—O3—C3—O3A	70.3 (3)	C3—O3—C6—C5	23.6 (6)
C6—O3—C3—C4	−5.4 (6)	O5—C5—C6—O3A	−125.5 (5)
C6—O3—C3—C2	112.1 (4)	C10—C5—C6—O3A	115.9 (5)
C6—O3A—C3—O3	−70.3 (3)	C4—C5—C6—O3A	−3.8 (5)
C6—O3A—C3—C4	40.3 (8)	O5—C5—C6—O3	−152.7 (3)
C6—O3A—C3—C2	146.2 (7)	C10—C5—C6—O3	88.7 (4)
O2—C2—C3—O3	155.5 (3)	C4—C5—C6—O3	−31.0 (4)
C1—C2—C3—O3	−94.4 (4)	C2—O2—C7—O1	−15.6 (3)
O2—C2—C3—O3A	172.8 (5)	C2—O2—C7—C8	−132.3 (3)
C1—C2—C3—O3A	−77.1 (5)	C2—O2—C7—C9	102.1 (3)
O2—C2—C3—C4	−84.8 (3)	C1—O1—C7—O2	1.4 (3)
C1—C2—C3—C4	25.3 (4)	C1—O1—C7—C8	118.0 (4)
C1—O4—C4—C3	17.3 (3)	C1—O1—C7—C9	−118.4 (3)
C1—O4—C4—C5	128.2 (3)	O7—N1—C10—C5	−104.9 (3)
O3—C3—C4—O4	99.7 (4)	O6—N1—C10—C5	74.5 (3)
O3A—C3—C4—O4	74.0 (4)	O5—C5—C10—N1	55.5 (3)
C2—C3—C4—O4	−26.1 (3)	C6—C5—C10—N1	175.3 (2)
O3—C3—C4—C5	−14.6 (5)	C4—C5—C10—N1	−72.4 (3)
O3A—C3—C4—C5	−40.3 (4)		

### Hydrogen-bond geometry (Å, °)

**Table:**

*D*—H···*A*	*D*—H	H···*A*	*D*···*A*	*D*—H···*A*
O5—H5···O4	0.86 (6)	2.13 (6)	2.696 (3)	123 (5)
O5—H5···O3^i^	0.86 (6)	2.24 (6)	2.703 (3)	114 (4)
C1—H1···O5^ii^	0.98	2.48	3.371 (3)	152
C4—H4···O6	0.98	2.39	3.074 (4)	126

Symmetry codes: (i) *x*+1, *y*, *z*; (ii) *x*−1/2, −*y*+1/2, −*z*+2.

## References

[bb1] Bruker (2007). *SMART* and *SAINT* Bruker AXS Inc., Madison, Wisconsin, USA.

[bb2] Choi, W. B., Wilson, L. J., Yeola, S., Liotta, D. C. & Schinazi, R. F. (1991). *J. Am. Chem. Soc.* **113**, 9377–9379.

[bb3] Ke, Y., Ji, X.-M., Zhu, Y. & Liu, H.-M. (2009). *Chin* *J. Struct. Chem.* **3**, 291–294.

[bb4] Kvaernø, L., Wightman, R. H. & Wengel, J. (2001). *J. Org. Chem.* A**66**, 5106–5112. 10.1021/jo015602v 11463263

[bb5] Liu, F.-W., Liu, H.-M., Ke, Y. & Zhang, J.-Y. (2004). *Carbohydr. Res.* **339**, 2651–2656. 10.1016/j.carres.2004.09.013 15519323

[bb6] Saito, Y., Zevaco, T. A. & Agrofoglio, L. A. (2002). *Tetrahedron*, **58**, 9593–9603.

[bb7] Sheldrick, G. M. (1996). *SADABS* University of Göttingen, Germany.

[bb8] Sheldrick, G. M. (2008). *Acta Cryst.* A**64**, 112–122. 10.1107/S0108767307043930 18156677

[bb9] Zhang, Q., Ke, Y., Cheng, W., Li, P. & Liu, H. (2011). *Acta Cryst.* E**67**, o1402. 10.1107/S1600536811017314 PMC3120445 21754787

[bb10] Zhang, J.-Y. & Yang, J. (2010). *Acta Cryst.* E**66**, o1704. 10.1107/S1600536810022774 PMC3007024 21587924

